# RuSentiTweet: a sentiment analysis dataset of general domain tweets in Russian

**DOI:** 10.7717/peerj-cs.1039

**Published:** 2022-07-19

**Authors:** Sergey Smetanin

**Affiliations:** Department of Business Informatics, Graduate School of Business, National Research University Higher School of Economics, Russia

**Keywords:** Sentiment dataset, Sentiment analysis, Russian

## Abstract

The Russian language is still not as well-resourced as English, especially in the field of sentiment analysis of Twitter content. Though several sentiment analysis datasets of tweets in Russia exist, they all are either automatically annotated or manually annotated by one annotator. Thus, there is no inter-annotator agreement, or annotation may be focused on a specific domain. In this article, we present RuSentiTweet, a new sentiment analysis dataset of general domain tweets in Russian. RuSentiTweet is currently the largest in its class for Russian, with 13,392 tweets manually annotated with moderate inter-rater agreement into five classes: Positive, Neutral, Negative, Speech Act, and Skip. As a source of data, we used Twitter Stream Grab, a historical collection of tweets obtained from the general Twitter API stream, which provides a 1% sample of the public tweets. Additionally, we released a RuBERT-based sentiment classification model that achieved *F*_1_ = 0.6594 on the test subset.

## Introduction

Recently, Twitter has been established as a major research platform, utilized in more than ten thousand research articles over the past ten years. Sentiment analysis has proven to be one of the major research areas (Antonakaki, Fragopoulou & Ioannidis, 2021). As expected, there is an interest in the sentiment analysis of the Russian-speaking segment of Twitter, not only for training machine learning (ML) models (Kotelnikova, 2020; Araslanov, Komotskiy & Agbozo, 2020; Kanev et al., 2022), but also for applied research—such as studying migration issues (Borodkina & Sibirev, 2019), measuring reactions to different events (Kirilenko & Stepchenkova, 2017; Kausar, Soosaimanickam & Nasar, 2021), and monitoring public sentiment (Chizhik, 2016; Smetanin, 2017). However, despite the fact that there are several datasets of tweets in Russian (Smetanin, 2020a), they are either annotated automatically (*e.g*., RuTweetCorp by Rubtsova (2013)) or annotated only by one annotator; thus, there is no inter-annotator agreement (*e.g*., Twitter Sentiment for 15 European Languages by Mozeticar & Smailović (2016)), or focused on a specific domain (*e.g*., SentiRuEval-2015 by Loukachevitch et al. (2015)). Thus, this research community lacks a general domain sentiment dataset of tweets in Russian that is annotated manually with reported inter-rater agreement score.

In this article, we present RuSentiTweet, a new sentiment analysis dataset of 13,392 general domain tweets in Russian. RuSentiTweet was annotated manually using RuSentiment guidelines (Rogers et al., 2018) into five classes (*Positive*, *Neutral*, *Negative*, *Speech Act*, and *Skip*) with moderate inter-rater agreement. The practical and academic contribution of this study is threefold. Firstly, we reviewed existing public sentiment dataset of tweets in Russian. Secondly, we filled the data gap and introduced RuSentiTweet, the only dataset of general domain tweets with manual annotation for the Russian language. Lastly, we trained several ML models to provide further research with a strong baseline.

The rest of the article is organized as follows. In “Related Work”, we review related research, identify existing public sentiment datasets of tweets in Russian, and confirm the importance of a new dataset of general domain tweets in Russian. In “Sentiment Dataset”, we describe the creation of RuSentiTweet. In “Sentiment Classification Baseline”, we document the training of several ML models to provide the research community with public baselines. In “Conclusion”, we present conclusions from this study.

## Related work

As of 2022, Russian was the eighth most widely-spoken language worldwide, with a total number of 258.2 million speakers (Szmigiera, 2022). Yet as reported in the preliminary results of the All-Russian Census 2020 (Rosstat, 2022), only about 147 million people permanently live in Russia. In addition to Russia, where Russian is the official language, it is also widely spoken in a number of other countries that were part of the USSR. According to various sources (Arefiev, 2013; Lopatin & Ulukhanov, 2017), there are from 52 to 94 million native speakers of the Russian language in these countries. A large number of Russian speakers also live in other countries such as those in Europe, the USA, Canada, Israel, and others (Lopatin & Ulukhanov, 2017). Given the significant Russian-speaking population and the ever-growing level of Internet penetration, texts published by Russian-speaking users on social networks are attracting more and more attention from researchers. As a result, every year new works appear both in the classical analysis of the sentiment of Russian-language content (*e.g*., Araslanov, Komotskiy & Agbozo, 2020; Kanev et al., 2022; Kausar, Soosaimanickam & Nasar, 2021) and in related areas, such as the identification of emotions (*e.g*., Babii, Kazyulina & Malafeev, 2020; Kazyulina, Babii & Malafeev, 2020; Babii, Kazyulina & Malafeev, 2021), toxicity and hate speech detection (*e.g*., Zueva, Kabirova & Kalaidin, 2020; Pronoza et al., 2021; Smetanin & Komarov, 2021b), and inappropriate language identification (*e.g*., Babakov et al., 2021; Babakov, Logacheva & Panchenko, 2022).

However, the Russian language is not as well-resourced as the English language (Besacier et al., 2014), especially in the field of sentiment analysis (Smetanin & Komarov, 2021a), so the data options for researchers are quite limited. In our previous study (Smetanin, 2020a), we identified 14 publicly available sentiment analysis datasets of Russian texts. In said study, we considered only those datasets that can be accessed *via* instructions from their original papers or official websites. Following this strategy, we omitted several existing datasets—such as ROMIP datasets (Chetviorkin, Braslavskiy & Loukachevich, 2013; Chetvirokin & Loukachevitch, 2013)—because we were unable to obtain access to them. Among these 14 datasets, only six datasets were constructed based on Twitter content, so we selected them for further detailed analysis. Additionally, we analysed the most recent review of sentiment analysis datasets of Russian texts by Kotelnikov (2021) but did not find any new Twitter datasets for consideration.

As can be seen from Table 1, RuTweetCorp (Rubtsova, 2013) is the largest sentiment analysis dataset of general domain tweets in Russian, but it was automatically annotated based on the strategy proposed by Read (2005): each tweet was assigned with the sentiment class based on the emoticons it contains. As a consequence, even a simple rule-based approach based on the presence of the ‘(’ character can achieve *F*_1_ = 97.39% in the binary (*Positive* and *Negative* classes) classification task (Smetanin & Komarov, 2021a). SemEval-2016 Task 5 Russian (Pontiki et al., 2016), SentiRuEval-2016 (Lukashevich & Rubtsova, 2016) and SentiRuEval-2015 (Loukachevitch et al., 2015) are manually annotated and widely used datasets, but they are all tied to a specific domain such as restaurants, automobiles, telecommunication companies, or banks. Twitter Sentiment for 15 European Languages (Mozeticar & Smailović, 2016) is a sentiment analysis dataset with manual annotation, but only one annotator was engaged for Russian-language tweets; thus, there is no inter-annotator agreement. The Kaggle dataset did not report data collection and annotation procedure. Thus, there is a lack of general domain sentiment dataset of tweets in Russian that is annotated manually with reported inter-rater agreement score.

**Table 1:** Sentiment analysis datasets of Russian language texts. More detailed description of each datasetcan be found in Smetanin (2020a), Smetanin & Komarov (2021a), Kotelnikov (2021), as well as in original papers (if published). For datasets that contain several subsets from different data sources, we indicated only those subsets that are made from tweets.

Dataset	Data source	Domain	Annotation	Classes	Size	Link
Twitter Sentiment for 15 European Languages (Mozeticar & Smailović, 2016)	Twitter	General	Manual	3	107,773	Project page
SemEval-2016 Task 5 Russian (Pontiki et al., 2016)	Twitter	Restaurants	Manual	3	405	Project page
SentiRuEval-2016 (Lukashevich & Rubtsova, 2016)	Twitter	Telecom and banks	Manual	3	23,595	Project page
SentiRuEval-2015 (Loukachevitch et al., 2015)	Twitter	Telecom and banks	Manual	4	16,318	Project page
RuTweetCorp (Rubtsova, 2013)	Twitter	General	Automatic	3	334,836	Project page
Kaggle Russian_twitter_sentiment	Twitter	n/a	n/a	2	226,832	Kaggle page

## Sentiment dataset

### Data collection

For a data source of tweets in Russian, we decided to use the Twitter Stream Grab (https://archive.org/details/twitterstream), a publicly available historical collection of JSON grabbed from the general Twitter “Spritzer” API stream. According to Twitter, this API provides a 1% sample of the complete public tweets and is not tied to a specific topic, so we considered it as a good source of general domain tweets. Additionally, several studies (Wang, Callan & Zheng, 2015; Leetaru, 2019) performed independent validation of the representativeness of this stream. Since the Twitter Stream Grab consists of tweets in different languages, our first step was to remove tweets written in non-Russian languages. Each tweet from this data source already contained information about the language of the text automatically detected^1^ 1Assessing the quality of a given algorithm lies outside the scope of this study. Initial research in this direction has already been done in other studies; for example, Pavliy and Lewis (2016) compared the quality of Twitter’s language detection algorithm and Google’s Compact Language Detector on Ukrainian and Russian tweets. The authors found that Twitter’s algorithm correctly detects 92% of texts in Russian and has higher accuracy than Google’s Compact Language Detector. by Twitter, so the language filtering procedure was fairly straightforward.

We downloaded the Twitter Stream Grab for 12 months from January 2020 to December 2020^2^ 2At the time of this writing, all months for 2021 were not available.. The main motivation for choosing an entire year as the interval was to cover all months of the year to minimize the effect of seasonality. Previous research has shown that there are daily (Larsen et al., 2015; Prata et al., 2016), weekly (Ten Thij, Bhulai & Kampstra, 2014; Dzogang, Lightman & Cristianini, 2017b), and seasonal (Dzogang et al., 2017a) patterns of sentiment or emotion expression on Twitter. Also, it has been found (Baylis et al., 2018; Baylis, 2020) that expressed sentiment correlates with weather, which also tends to depend on the season. After excluding retweets and filtering by language, we obtained ∼4.5M tweets in Russian. Since manual labelling of such a volume of tweets is costly and extremely time-consuming, we randomly selected 15,000 tweets for further annotation (tweets evenly distributed over the selected months).

### Data annotation

#### Guidelines

As per recommendations outlined in our previous study (Smetanin, 2020a), we decided to use RuSentiment (Rogers et al., 2018) annotation guidelines (https://github.com/text-machine-lab/rusentiment/tree/master/Guidelines). To the best of our knowledge, this is the only set of publicly available sentiment annotation guidelines designed for the Russian language. The guidelines are described in detail in the original RuSentiment paper, so this section provides only key summary.

The annotation guidelines cover both implicit and explicit forms of expressions for external attitude (evaluation) and the internal emotional state (mood). The guidelines cover five sentiment classes.

- *Negative* represents both explicit and implicit negative sentiment or attitude towards something.
- *Neutral* represents texts that simply describe some situation in a neutral, matter-of-fact way and have no clear positive or negative sentiment. This class also includes commercial information, factual questions, objective descriptions, and summaries.
- *Positive* represents both explicit and implicit positive sentiment or attitude towards something.
- *Speech Act* represents texts that perform the functions of various speech acts—such as greeting someone, congratulating someone, and expressing gratitude for something. Although these texts also represent a positive sentiment, they are treated as a separate subcategory because they can also be performed under social pressure or out of a feeling of obligation (Rogers et al., 2018).
- *Skip* represents noisy and unclear sentiment or attitude towards something—such as when the original meaning is impossible to ascertain without additional context, the sentiment of the texts as a whole is not entirely clear, the text is not in Russian, or the text contains jokes.

Text with irony was annotated with the dominant sentiment, commonly negative. Hashtags were treated as information units similar to basic words or phrases. Emoticons were not treated as the only sentiment labels but were analysed in combination with the whole text to identify dominant sentiment.

#### Crowdsourcing platform

**Figure 1:**
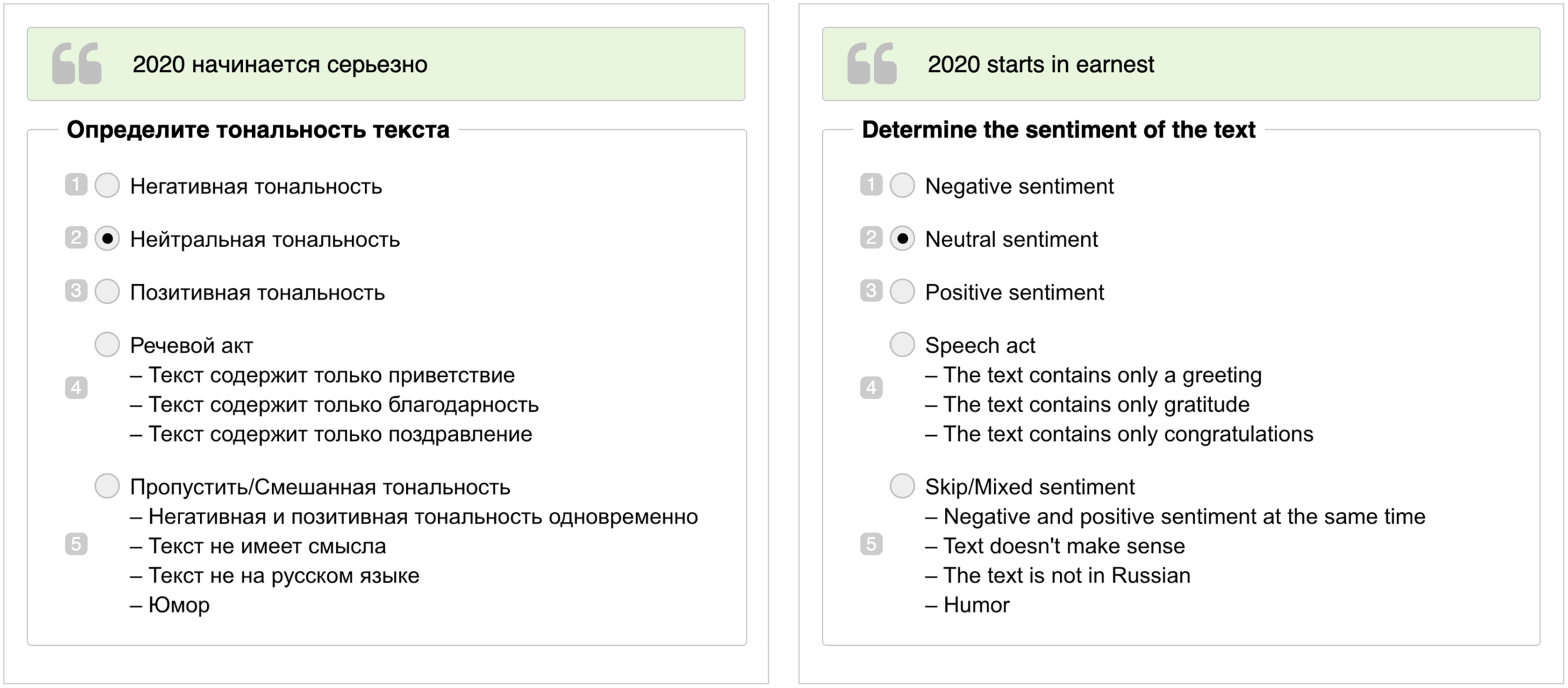
An example of user interface for annotators in Yandex.Toloka in Russian (on the left) and its translation in English (on the right). The green block with quotation marks contains the text of the tweet. Under the block with the text, there are numbered sentiment classes, where 1 is *Negative*, 2 is *Neutral*, 3 is *Positive*, 4 is *Speech Act*, and 5 is *Skip*. Numbers are used as hotkeys during annotation.

The annotation was performed *via* Yandex.Toloka (https://toloka.ai/), a Russian crowd-sourcing platform with a high share of Russian speaking workers. Yandex.Toloka is widely used in the studies on Russian-language content, such as for annotation of semantic change (Rodina & Kutuzov, 2020), question answering (Korablinov & Braslavski, 2020), and toxic comments (Smetanin, 2020b). A depiction of the Yandex.Toloka user interface can be found in Fig. 1. We required annotators to pass training before starting annotation. During the annotation of the dataset, their work was continuously evaluated through honeypots. As training samples and control pairs, we selected texts from RuSentiment, which was annotated using the same guidelines. The threshold was 60% correctly annotated samples for training and 80% samples for honeypots. We selected only Russian speaking annotators who passed an internal exam (https://toloka.ai/ru/docs/guide/concepts/filters.html) on language knowledge.

#### Aggregation

Following the RuSentiment aggregation strategy, a tweet was deemed to belong to a class if at least two out of the three annotators attributed it to that class. In case all three annotators disagreed, the tweet was removed from the dataset as extremely noisy and unclear (see examples in Table A1). Out of the initially selected 15,000 tweets, 1,608 tweets received all three different annotations, so we excluded these tweets from the final dataset. Thus, the final dataset consists of 13,392 tweets with the following class distribution: 3,298 (24.62%) *Negative* tweets, 5,341 (39.88%) *Neutral* tweets, 2,414 (18.02%) *Positive* tweets, 1,843 (13.76%) *Skip* tweets, and 496 (3.70%) *Speech Act* tweets. We split our dataset into training (80%) and test subset (20%) using stratified random sampling by class labels.

#### Inter-annotator agreement

For measuring the inter-annotator agreement, we calculated the Krippendorff’s *α* coefficient (Krippendorff, 1980) because it applies to any number of annotators and categories, as well as to missing or incomplete data (Krippendorff, 2004). For most inter-annotator agreement indices, including Krippendorff’s *α*, it is commonly suggested that a cutoff threshold value of 0.8 is a marker of good reliability, with a range of 0.667 to 0.8 allowing for tentative conclusions and values below 0.667 indicating poor agreement (Beckler et al., 2018). However, in the systematic review of crowd-sourced annotation in social computing, Salminen et al. (2018) reported that agreement scores in social computing studies are not high, averaging at around 0.60 for both Kappa and Alpha metrics, which is lower than typical threshold values. The authors highlighted that the nature of annotation in social computing tends to be more subjective rather than objective, and the more subjective the task, the worse the agreement, regardless of the metric used. Though it is important to report inter-rater agreement scores, there are suggestions that the results can be misleading in social computing (Hillaire, 2021). In fact, low agreement in this case does not necessarily mean the opinions of annotators are incorrect; it may simply indicate that they have different opinions (Salminen et al., 2018; Hillaire et al., 2021). Sentiment annotation, by nature, is a subjective task because the annotator must subjectively (with some guidelines) identify sentiment and emotions expressed by the author and not just objectively analyse narrated events or situations: we can expect annotators to have different subjective understanding of emotion expressed in a particular text. Thus, considering that in the field of social computing science the mean score is 0.60 (Salminen et al., 2018), we followed the same approach as Hillaire (2021) and adopted the less conservative interpretation of inter-rater agreement by Landis & Koch (1977), which suggests the following interpretations.

- Scores from 0.0 to 0.2 indicate a slight agreement.
- Scores from 0.21 to 0.40 indicate a fair agreement.
- Scores from 0.41 to 0.60 indicate a moderate agreement.
- Scores from 0.61 to 0.80 indicate a substantial agreement.
- Scores from 0.81 to 1.0 indicate almost perfect or perfect agreement.

We calculated Krippendorff’s *α* with binary distance (*e.g*., all classes have similar distance between each other) using the NLTK library (Bird, Klein & Loper, 2009) and obtained the score of 0.5048 for binary distance, which can be interpreted as a moderate agreement between annotators. We considered this level of agreement as satisfactory for our case, since other five-class sentiment datasets also reported this or even lower level of agreement, such as Blog Track at TREC 2008 (*α* = 0.4219, five classes) (Bermingham & Smeaton, 2009), LINIS Crowd (*α* = 0.541, five classes) (Koltsova, Alexeeva & Kolcov, 2016), RuSentiment (Fleiss’ kappa of 0.58, five classes) (Rogers et al., 2018), sentiment@USNavy (*α* = 0.592, four classes) (Fiok et al., 2021), and NaijaSenti (Fleiss kappa of (0.434, 0.555), five classes) (Muhammad et al., 2022). Additionally, we calculated Krippendorff’s *α* with interval distance that takes into account distance between classes: for example, *Neutral* and *Positive* classes are closer to each other than *Negative* and *Positive* classes. The distance matrix is presented in Table 2. The Krippendorff’s *α* coefficient for interval distance was 0.5601, which can also be interpreted as slightly higher but still moderate agreement.

**Table 2:** Distance between classes for interval Krippendorff’s $\alpha$, where 0 means that classes are the same, 1 means that classes are close to each other, and 2 means that classes a far away from each other.

Class	Negative	Neutral	Positive	Speech	Skip
Negative	0	1	2	2	1
Neutral	1	0	1	1	1
Positive	2	1	0	0	1
Speech	2	1	0	0	1
Skip	1	1	1	1	0

**Note:**

*Positive* and *Speech* classes have zero distance between them; they both represent positive sentiment as per RuSentiment guidelines.

### Explanatory analysis

**Figure 2:**
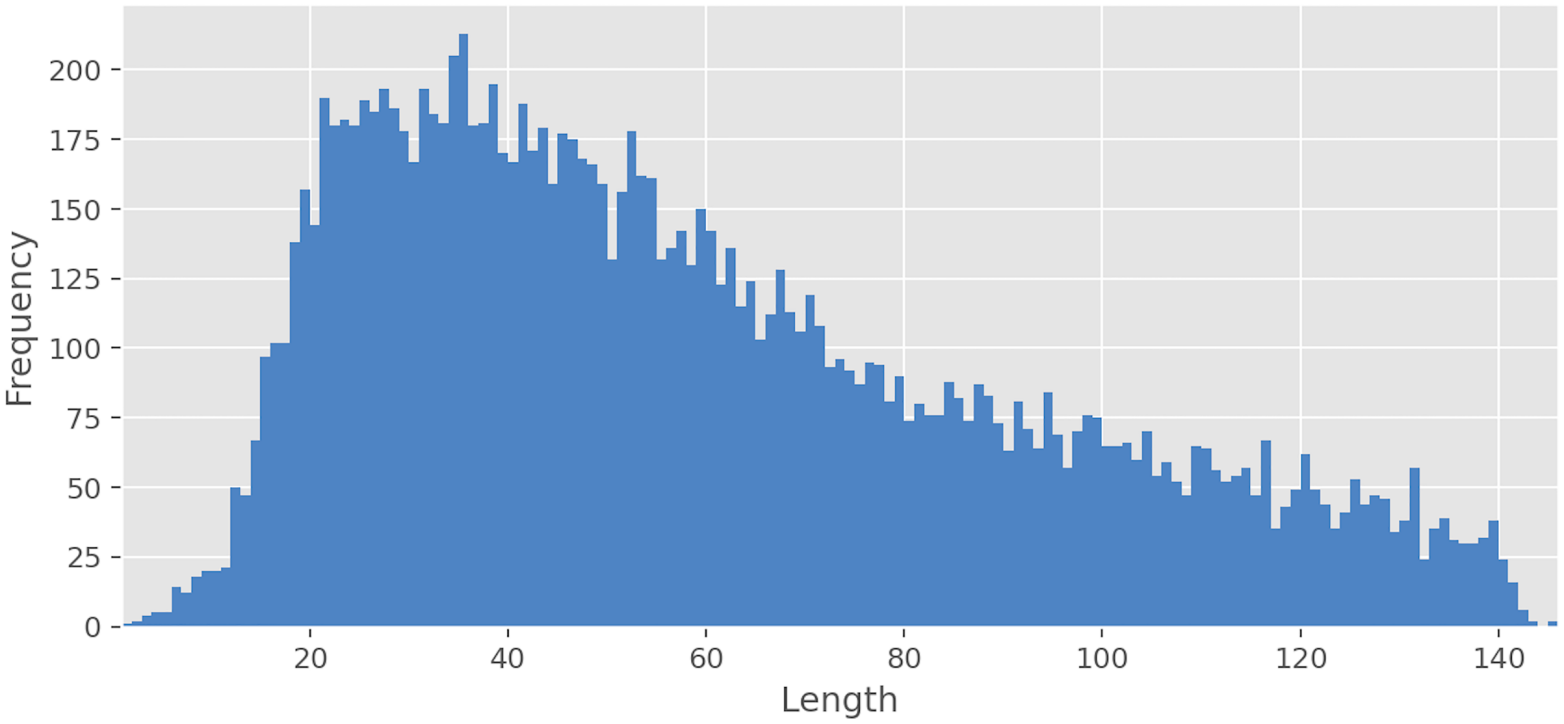
Texts length distribution.

The average text length is 59.36 characters for all text, 67.52 for *Negative*, 59.29 for *Neutral*, 57.85 for *Positive*, 42.71 for *Speech*, and 51.41 for *Skip*. As can be seen from Fig. 2, the frequency of occurrence of texts from a pair of characters in the dataset is extremely low, but with an increase in the number of characters, rapid growth begins. The frequency peak is reached when the text length is from 20 to 40 characters, and then the frequency gradually begins to decrease. Interestingly, for some classes, there is a moderate Pearson’s correlation between the length of the text and the proportion of texts with this class relative to all texts. The *Negative* class has a moderate positive correlation (*ρ* = 0.68, *p* < 0.01) with the length of text, whereas *Speech* (*ρ* = −0.52, *p* < 0.01) and *Skip* (*ρ* = −0.62, *p* < 0.01) classes have moderate negative correlation. At the same time, *Neutral* (*ρ* = −0.03, *p* = 0.70) and *Positive* (*ρ* = −0.04, *p* = 0.62) classes do not have statistically significant correlation. The most common unigrams, bigrams, and emojis can be found in Table 3.

**Table 3:** Most common unigrams, bigrams, and emojis without stop words, punctuation, and numbers. Stop words were removed using NLTK (Bird, Klein & Loper, 2009). Most unigrams and bigrams can have several English translations depending on the context. The table provides only one translation option.

Unigram			Bigram			Emoji	
Item		Count	Item		Count	Item	Count
Russian	English		Russian	English			
это	it	1,117	доброе утро	good morning	39	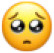	443
просто	simply	355	спокойной ночи	good night	26	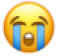	313
спасибо	thanks	306	спасибо большое	thanks a lot	24	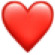	246
хочу	want	249	самом деле	actually	23	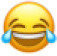	240
ещё	yet	223	это просто	it’s simple	23	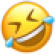	120
почему	why	209	опубликовано фото	published photo	18	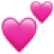	119
очень	very	205	сих пор	so far	17	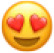	118
всё	all	204	руб г	rub g	16	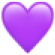	113
блять	fuck	184	днем рождения	birthday	15	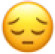	104
вообще	generally	174	все ещё	still	13	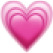	100

As mentioned in “Related Work”, one of the key limitations of RuTweetCorp (Rubtsova, 2013)—the biggest automatically annotated dataset of tweets in Russian—is that *Positive* and *Negative* tweets in it can be easily separated with *F*_1_ = 97.39% by a simple rule-based approach based on the presence of the ‘(’ character. We decided to check that this limitation does not apply to RuSentiTweet. We applied this simple rule-based approach to *Positive* and *Negative* tweets from RuSentiTweet and got *F*_1_ = 0.3450 (*i.e*., approximately the same result as in the case of a random classification), thereby confirming that RuTweetCorp’s limitation does not apply to RuSentiTweet.

## Sentiment classification baseline

### Model selection

As was demonstrated in our recent study (Smetanin & Komarov, 2021a), sentiment analysis of the Russian language text based on the language models tends to outperform rule-based and basic ML-based approaches in terms of classification quality. This statement was also supported by other studies (Golubev & Loukachevitch, 2020; Kotelnikova, 2020; Konstantinov, Moshkin & Yarushkina, 2021). Based on the mentioned papers, we decided to fine-tune RuBERT (Kuratov & Arkhipov, 2019), a version of BERT (Devlin et al., 2019) trained on the Russian part of Wikipedia and Russian news. Over the past few years, this model has been actively used in sentiment analysis studies on the Russian language and constantly demonstrated strong or even new state-of-the-art (SOTA) results (Golubev & Loukachevitch, 2020; Kotelnikova, 2020; Konstantinov, Moshkin & Yarushkina, 2021; Smetanin & Komarov, 2021a). For comparison, we also decided to train more a classical ML classifier for sentiment analysis task: Multinomial Naive Bayes (MNB). We used the MNB implementation (https://github.com/sismetanin/sentiment-analysis-of-tweets-in-russian) from our previous paper (Smetanin & Komarov, 2019).

### Results

During the training stage for RuBERT, we relied on the approach used in Smetanin & Komarov (2021a). Fine-tuning was performed using the Transformers library (Wolf et al., 2020) on 1 Tesla V100 SXM2 32GB GPU with the following parameters: four train epochs, 128 max sequence length, 32 batch size, and a learning rate of 5e−5. Since our goal was to provide a baseline classification model and not the most efficient one, we did not search for the most efficient training parameters. We repeated each experiment 3 times and reported mean values of the measurements. For MNB, we used the same parameters as in our previous paper (Smetanin & Komarov, 2019): combination of unigrams and bigrams, TF-IDF vectorizer, and an alpha of 0.01.

According to the results presented in Table 4, RuBERT outperformed MNB, as expected, and showed the best classification scores. The classification results obtained on RuSentiTweet are slightly lower but still comparable with the results obtained in other studies on RuSentiment (see Table 5): RuBERT achieved $F_1^{weighted} = 0.7263$ on RuSentiment (Kuratov & Arkhipov, 2019), whereas on our dataset this model showed $F_1^{weighted} = 0.6675$. The difference in the results could be caused by the size of the dataset because RuSentiment is more than two times bigger. The classification metrics of five-class sentiment analysis approaches on other datasets in other languages can be found in Table 5. Although direct comparison for different datasets and languages may not be entirely correct, we can see that at least the magnitude of order of our approach corresponds with the average score for five-class classification.

**Table 4:** Five-class sentiment classification on RuSentiTweet.

Model	Precision	Recall	${\bf F}_{\bf 1}^{{\bf macro}}$	${\bf F}_{\bf 1}^{{\bf weighted}}$
RuBERT	0.6793	0.6449	0.6594	0.6675
MNB	0.5867	0.5021	0.5216	0.5189

**Table 5:** Five-class sentiment classification studies.

Study	Dataset	Model	Classification metrics				
			Accuracy	Precision	Recall	${\bf F}_{\bf 1}^{{\bf macro}}$	${\bf F}_{\bf 1}^{{\bf weighted}}$
Muhammad et al. (2022)	NaijaSenti	XLM-R-base+LAFT	n/a	n/a	n/a	n/a	0.795
Muhammad et al. (2022)	NaijaSenti	M-BERT+LAFT	n/a	n/a	n/a	n/a	0.7700
Fiok et al. (2021)	sentiment@USNavy	BART large + CNN	n/a	n/a	n/a	0.596	n/a
Smetanin & Komarov (2021a)	RuSentiment	M-BERT-Base	n/a	0.6722	0.6907	0.6794	0.7244
Smetanin & Komarov (2021a)	RuSentiment	RuBERT	n/a	0.7089	0.7362	0.7203	0.7571
Smetanin & Komarov (2021a)	RuSentiment	M-USE-CNN	n/a	0.6571	0.6708	0.6627	0.7105
Smetanin & Komarov (2021a)	RuSentiment	M-USE-Trans	n/a	0.6821	0.6982	0.6860	0.7342
Jamadi Khiabani, Basiri & Rastegari (2020)	TripAdvisor	Dempster–Shafer-based model	0.79	0.5	0.47	0.49	n/a
Jamadi Khiabani, Basiri & Rastegari (2020)	CitySearch	Dempster–Shafer-based model	0.79	0.48	0.48	0.48	n/a
Kuratov & Arkhipov (2019)	RuSentiment	Multilingual BERT	n/a	n/a	n/a	n/a	0.7082
Kuratov & Arkhipov (2019)	RuSentiment	RuBERT	n/a	n/a	n/a	n/a	0.7263
Baymurzina, Kuznetsov & Burtsev (2019)	RuSentiment	SWCNN + fastText Twitter	n/a	n/a	n/a	n/a	0.7850
Baymurzina, Kuznetsov & Burtsev (2019)	RuSentiment	BiGRU + ELMo Wiki	n/a	n/a	n/a	n/a	0.6947
Tripto & Ali (2018)	YouTube	LSTM	0.5424	n/a	n/a	0.5320	n/a
Li et al. (2018)	Twitter	Logistic Regression	0.6899	0.6053	0.6899	0.6354	n/a
Ahmadi et al. (2017)	SST-5	RNTN	0.41	n/a	n/a	0.32	n/a
Buntoro, Adji & Purnamasari (2016)	Twitter	Naïve Bayes	0.7177	0.716	0.718	n/a	n/a
Aly & Atiya (2013)	LABR	SVM	0.503	n/a	n/a	n/a	0.491
Chetvirokin & Loukachevitch (2013)	ROMIP-2012 (Movies)	n/a	0.407	n/a	n/a	0.377	n/a
Blinov, Kotelnikov & Pestov (2013)	ROMIP-2012 (Books)	SVM	0.481	0.339	0.496	0.402	n/a
Chetvirokin & Loukachevitch (2013)	ROMIP-2012 (Cameras)	n/a	0.480	n/a	n/a	0.336	n/a
Pak & Paroubek (2012)	ROMIP-2011 (Movies)	SVM	0.599	n/a	n/a	0.286	n/a
Pak & Paroubek (2012)	ROMIP-2011 (Books)	SVM	0.622	n/a	n/a	0.291	n/a
Pak & Paroubek (2012)	ROMIP-2011 (Cameras)	SVM	0.626	n/a	n/a	0.342	n/a

**Note:**

We selected only those studies, which consideredfive sentiment classes and reported at least one of the following classification measures: Precision, Recall, macro *F1*, weighted *F1*. Among all datasets, only ROMIP (Chetviorkin, Braslavskiy & Loukachevich, 2013; Chetvirokin & Loukachevitch, 2013) and RuSentiment (Rogers et al., 2018) datasets are in Russian.

We made our RuBERT-based model publicly available (https://huggingface.co/sismetanin/rubert-rusentitweet) to the research community.

### Error analysis

**Figure 3:**
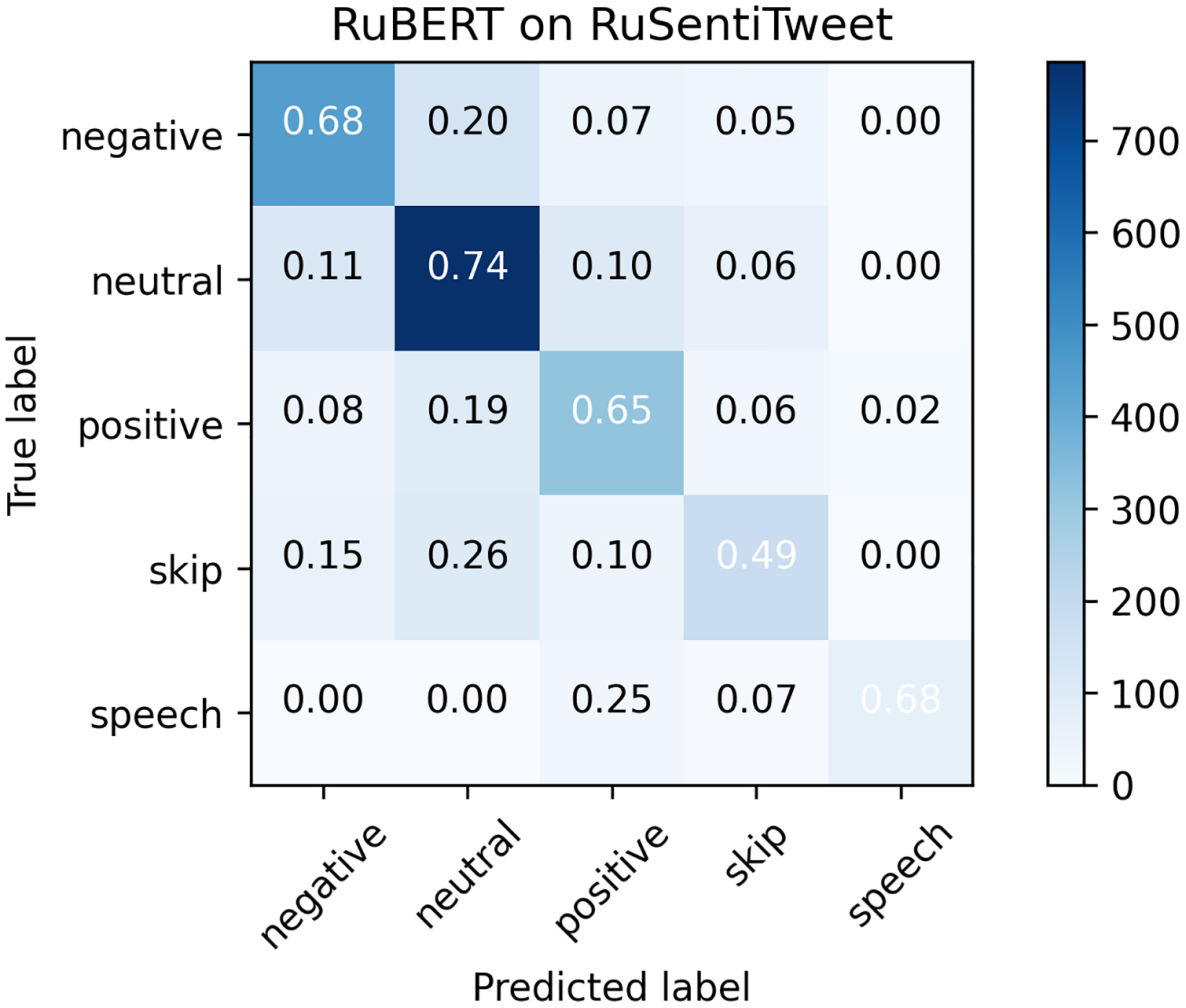
Confusion matrix for RuSentiTweet.

**Figure 4:**
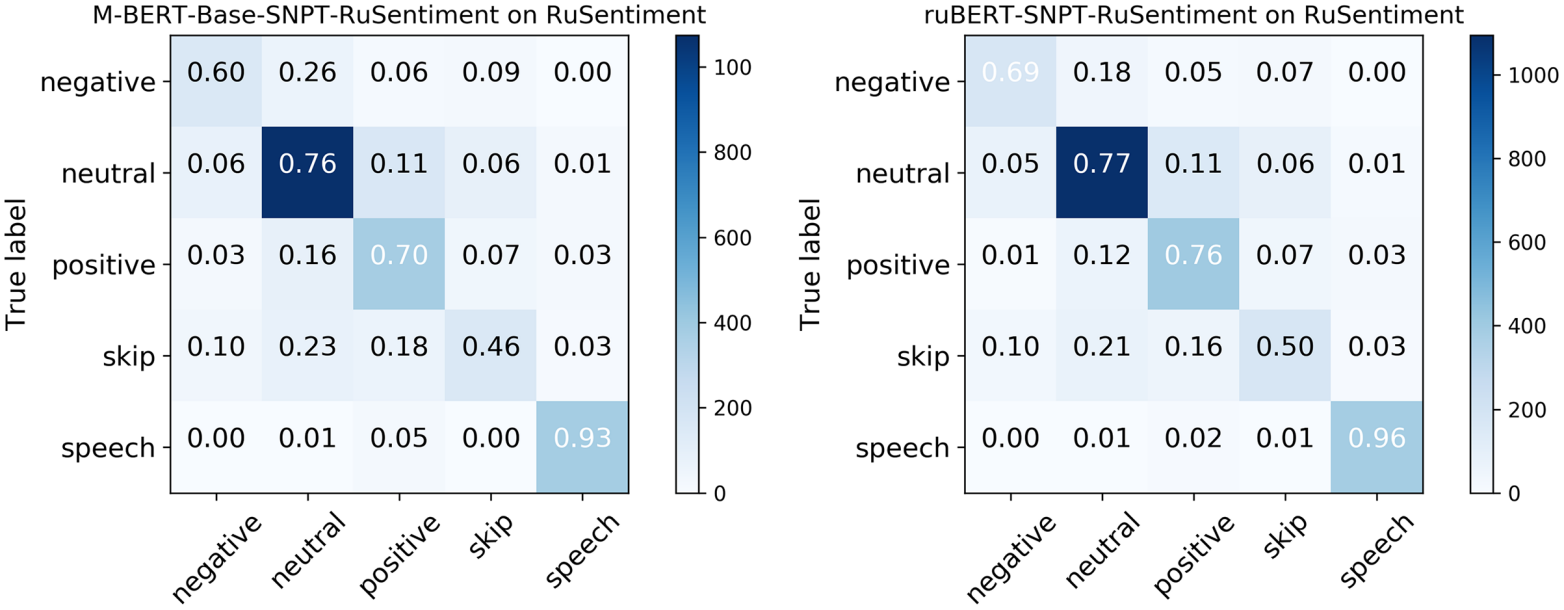
Confusion matrix for RuSentiment were created using molders from Smetanin & Komarov (2021a).

Considering that RuBERT clearly outperformed MNB, we performed error analysis only for RuBERT. As can be seen from confusion matrix for RuBERT (see Fig. 3), the *Skip* class was one of the most scarcely classified classes since it initially consisted of barely interpretable and noisy tweets. The *Speech Acts* class was clearly distinguished from *Negative* and *Neutral* classes because it consists of a well-defined group of speech constructs, but it was commonly misclassified as *Positive* because it also represents positive sentiment. Predictably, the *Neutral* class was commonly misclassified as *Positive* or *Negative* class because neutral sentiment is logically located between positive and negative sentiment. As was highlighted by Barnes, Øvrelid & Velldal (2019), the issue of neutral sentiment misclassification tends to be a general challenge of non-binary sentiment classification. In general, misclassification errors of our model were quite similar to RuSentiment misclassification errors reported in our previous study (Smetanin & Komarov, 2021a) (see Fig. 4), most likely because the same annotation guidelines and models were used. The most noticeable difference was in the recall for the *Speech* class. For RuSentiment, it was much better separated from other classes, with recall in the interval from 0.88 to 0.96 (Smetanin & Komarov, 2021a). We suppose that the reason of such a difference is in the number of texts in this class: RuSentiment contains 3,467 texts of the *Speech*, whereas RuSentiTweet contains only 480 such texts. The examples of misclassified tweets can be found in Table A2.

## Conclusion

In this article, we present RuSentiTweet, a new general domain sentiment dataset of tweets in Russian with manual annotation. RuSentiTweet includes 13,392 tweets annotated by three annotators with moderate inter-rater agreement into five classes: *Positive*, *Neutral*, *Negative*, *Speech Act*, and *Skip*. Currently, RuSentiTweet is the only dataset of general domain tweets in Russian with manual annotation by more than one annotator and is the largest in its class for Russian. Additionally, we presented a RuBERT-based model trained on RuSentiTweet, which demonstrated *F*_1_ = 0.6594 in five-class classification. The code, data, and model were made publicly available to the research community.

Further research might focus on several areas. Firstly, considerably more work must be done to determine the most efficient ML algorithm in terms of classification quality for RuSentiTweet. In particular, it could be interesting to apply explainable sentiment analysis approaches (*e.g*., Szczepański et al., 2021; Kumar & Raman, 2022) to allow a deeper understanding of the reasons for misclassification errors on particular texts. Secondly, it would be interesting to measure a subjective well-being index based on historical Russian tweets. Lastly, another possible area of future research would be to perform additional toxicity annotation of negative tweets from RuSentiTweet.

## Supplemental Information

10.7717/peerj-cs.1039/supp-1 Supplemental Information 1 Ref Heart Emoji.

10.7717/peerj-cs.1039/supp-2 Supplemental Information 2 Text Length Distribution.

10.7717/peerj-cs.1039/supp-3 Supplemental Information 3 Smiling Face with Heart Eyes Emoji.

10.7717/peerj-cs.1039/supp-4 Supplemental Information 4 Pleading Face Emoji.

10.7717/peerj-cs.1039/supp-5 Supplemental Information 5 Growing Heart Emoji.

10.7717/peerj-cs.1039/supp-6 Supplemental Information 6 Purple Heart Emoji.

10.7717/peerj-cs.1039/supp-7 Supplemental Information 7 Rolling on the Floor Laughing Emoji.

10.7717/peerj-cs.1039/supp-8 Supplemental Information 8 Loudly Crying Face Emoji.

10.7717/peerj-cs.1039/supp-9 Supplemental Information 9 Face With Tears of Joy Emoji.

10.7717/peerj-cs.1039/supp-10 Supplemental Information 10 Two Hearts Emoji.

10.7717/peerj-cs.1039/supp-11 Supplemental Information 11 Pensive Face Emoji.

## Figures and Tables

**Table A1:** Examples of tweets with no agreement between annotators.

Tweet		Annotation		
Russian	English	Annotator 1	Annotator 2	Annotator 3
ю ноу блин	yu know damn it	Skip	Neutral	Negative
@USER доброе утро всем дэдди сегодня	@USER good morning daddies to everyone today	Speech	Positive	Skip
Путешествуем по Уэльсу. не уважать мужчин	Traveling in Wales. disrespect men	Negative	Neutral	Skip
тот факт что в энимал кроссинге так мало прикольных мышиных жителей	the fact that there are so few funny mouse inhabitants in animal crossing	Negative	Positive	Neutral
Кто в нижнем родился, в верхнем не сгодился.	Who was born in the bottom, did not fit in the top.	Negative	Neutral	Skip

**Table A2:** Examples of tweets classification. All usernames and URLs were replaced with keywords for anonymity purposes.

Tweet		True class	Predicted class
Russian	English		
@USERNAME @USERNAME @USERNAME @USERNAME @USERNAME Помедорус	@USERNAME @USERNAME @USERNAME @USERNAME @USERNAME Pomedorus	Skip	Skip
@USERNAME ты не лохушка ЛОЛ я тебе завидую…. у меня травма из за интернета вот я лохушка	@USERNAME you’re not a sucker LOL I envy you…. I’m traumatized because of the internet I’m a sucker	Skip	Negative
@USERNAME Котиков Одриосолу Дождь	@USERNAME Cats Odriosolu Rain	Skip	Neutral
@USERNAME Уж лучше твоя грудь	@USERNAME Your breasts are better	Skip	Positive
Как сережки URL	How do you like the earrings URL	Neutral	Neutral
@USERNAME Реквием по мечте	@USERNAME Requiem for a dream	Neutral	Positive
@USERNAME ПОДОЖДИ НУ МНЕ КАЗАЛОСЬ ДА	@USERNAME WAIT I THINK YES	Neutral	Negative
@USERNAME Спокойной ночи и сладких снов	@USERNAME Good night and sweet dreams	Speech	Speech
@USERNAME как дела моя хорошая?? ( )	@USERNAME how are you my dear?? ( )	Speech	Positive
@USERNAME Это классно что у тебя есть эти люди	@USERNAME It’s great that you have these people	Positive	Positive
На самом деле я ловлю уруру с этого облачка.	In fact, I catch ururu from this cloud.	Positive	Neutral
@USERNAME Это классно что у тебя есть эти люди	What kind of morons are you, many have a school/work day tomorrow	Negative	Negative
интересный факт: смысла в клипах тхт больше, чем в твоей жизни	Interesting fact: there is more sense in txt clips than in your life	Negative	Neutral

## Additional Information and Declarations

### Data Availability

The following information was supplied regarding data availability: The code and dataset are available at GitHub: https://github.com/sismetanin/rusentitweet. The sentiment classification model is available at HuggingFace: https://huggingface.co/sismetanin/rubert-rusentitweet.
