# Supplementary figures and images for: RuSentiTweet: a sentiment analysis dataset of general domain tweets in Russian
Source: PeerJ Comput Sci. 2022 Jul 19;8:e1039. doi: 10.7717/peerj-cs.1039 (PMC9454938; doi:10.7717/peerj-cs.1039)

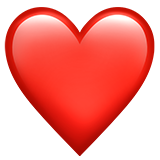

Supplement: Supplemental Information 1 [file peerj-cs-08-1039-s001.png]

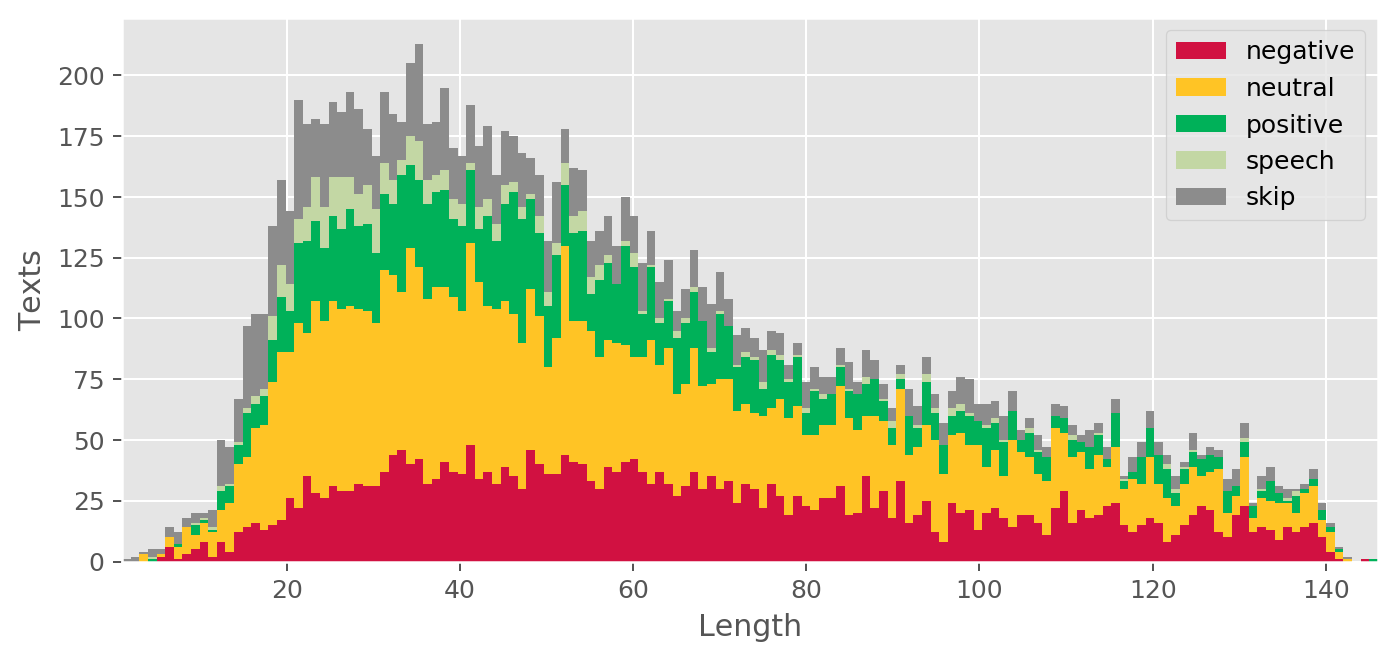

Supplement: Supplemental Information 2 [file peerj-cs-08-1039-s002.png]

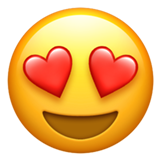

Supplement: Supplemental Information 3 [file peerj-cs-08-1039-s003.png]

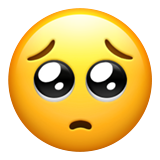

Supplement: Supplemental Information 4 [file peerj-cs-08-1039-s004.png]

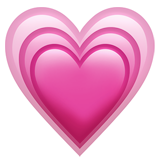

Supplement: Supplemental Information 5 [file peerj-cs-08-1039-s005.png]

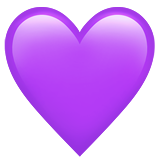

Supplement: Supplemental Information 6 [file peerj-cs-08-1039-s006.png]

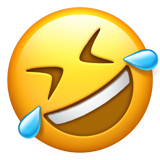

Supplement: Supplemental Information 7 [file peerj-cs-08-1039-s007.png]

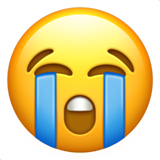

Supplement: Supplemental Information 8 [file peerj-cs-08-1039-s008.png]

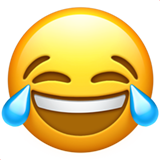

Supplement: Supplemental Information 9 [file peerj-cs-08-1039-s009.png]

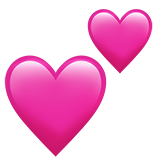

Supplement: Supplemental Information 10 [file peerj-cs-08-1039-s010.png]

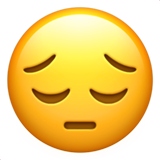

Supplement: Supplemental Information 11 [file peerj-cs-08-1039-s011.png]
